# Triglyceride glucose index is associated with functional coronary artery stenosis in hypertensive patients

**DOI:** 10.3389/fendo.2024.1323722

**Published:** 2024-03-25

**Authors:** Shuting Wang, Zhenzhou Shi, Hong Pan, Tiancai Yan, Ling Liu, Jiaheng Xu, Wei Wang, Tong Zhang

**Affiliations:** ^1^ Department of Radiology, The Fourth Affiliated Hospital of Harbin Medical University, Harbin, Heilongjiang, China; ^2^ The Magnetic Resonance Imaging Room, First Affiliated Hospital of Harbin Medical University, Harbin, Heilongjiang, China

**Keywords:** TyG index, fat attenuation index, CT-derived fractional flow reserve, hypertension, insulin resistance, computed tomography coronary angiography

## Abstract

**Background:**

The triglyceride glucose (TyG) index is an effective method for determining insulin resistance (IR). Limited research has explored the connection between the TyG index and functionally significant stenosis in hypertensive patients. Furthermore, the connections between the TyG index, fat attenuation index (FAI) and atherosclerotic plaque characteristics are also worth exploring.

**Methods:**

The study screened 1622 hypertensive participants without coronary artery disease history who underwent coronary computed tomography angiography. The TyG index was calculated as ln (fasting glucose [mg/dL] * fasting TG [mg/dL]/2). Adverse plaque characteristics (HRPCs), high-risk plaques (HRPs), FAI, and CT-derived fractional flow reserve (FFR_CT_) were analyzed and measured for all patients. Functionally significant stenosis causing ischemia is defined as FFR_CT_ ≤ 0.80. Two patient groups were created based on the FFR_CT_: the FFR_CT_ < 0.80 group and the FFR_CT_ > 0.80 group. In hypertensive patients, the association between the TyG index and FFR_CT_ was examined applying a logistic regression model.

**Results:**

The TyG index was higher for people with FFR_CT_ ≤ 0.80 contrast to those with FFR_CT_ > 0.80. After controlling for additional confounding factors, the logistic regression model revealed a clear connection between the TyG index and FFR_CT_ ≤ 0.80 (OR = 1.718, 95% CI 1.097–2.690, *p* = 0.018). The restricted cubic spline analysis displayed a nonlinear connection between the TyG index and FFR_CT_ ≤ 0.80 (*p* for nonlinear = 0.001). The TyG index increased the fraction of individuals with HRPs and HRPCs, FAI raised, and FFR_CT_ decreased (*p* < 0.05). The multivariate linear regression analysis illustrated a powerfulcorrelation between high TyG index levels and FAI, FFR_CT_, positive remodeling (PR), and low-attenuation plaque (LAPs) (standardized regression coefficients: 0.029 [*p* = 0.007], -0.051 [*p* < 0.001], 0.029 [*p* = 0.027], and 0.026 [*p* = 0.046], separately).

**Conclusion:**

In hypertensive patients, the TyG index showed an excellent association with a risk of FFR_CT_ ≤ 0.80. Additionally, the TyG index was also linked to FAI, FFR_CT_, PR, and LAPs.

## Introduction

A major public health concern, hypertension has a morbidity incidence of 23.2% in China (1). A significant predictor of atherosclerosis is hypertension, which often coexists with coronary heart disease (CHD) (2). Coronary artery disease (CAD) is more common in those with hypertension. However, a sizable fraction of individuals do not have CAD (3). In addition, studies have shown that disconnection between the presence of ischemia and the degree of coronary artery stenosis (CS) is common in CHD subjects. Only approximately half of obstructive lesions will lead to ischemia (4). Consequently, developing a low-cost, reliable, and broadly applicable biomarker to identify myocardial ischemia in hypertensive patients is crucial.

The seriousness and type of any coronary artery atherosclerosis, as well as the coronary anatomy, may all be assessed by computed tomography coronary angiography (CCTA) (5). CT-derived fractional flow reserve (FFR_CT_) can be used to accurately locate lesions that lead to decreased blood flow function and detect lesion-specific ischemia, and the gold standard for invasive coronary angiography (ICA) with fractional flow reserve (FFR) has confirmed it (6, 7). Functionally significant stenosis causing ischemia with significant hemodynamic changes is defined as FFR_CT_ ≤ 0.80 at each patient level (8, 9). An FFR threshold of 0.80 helps identify people who can benefit from coronary revascularization, according to several randomized trials. FFR is a commonly utilized metric for figuring out the functional value of CAD in a lesion-specific way (10).

The triglyceride glucose (TyG) index has been put forward as a simple and economical method to assess insulin resistance (IR) (11). TyG index and the following variables have been proven to be significantly correlated in numerous studies: the prevalence of cardiovascular disease, myocardial infarction risk, the prognosis for chronic heart failure, and in-stent stenosis (12–14). The heightened TyG index is a notable independent risk factor for coronary heart disease, linked to a higher chance of major adverse cardiovascular and cerebral events (15, 16). Additionally, there is a correlation between the rising TyG index and the increasing prevalence of hypertension, and the TyG index can be useful for forecasting the potential for hypertension in ordinary people (17, 18). Our previous investigation revealed an excellent correlation between the TyG index and CS > 50% in hypertensive subjects (3). The TyG index can be applied to forecast the adverse consequences for individuals with coronary heart disease or hypertension (2). However, the current study is limited to exploring the connection between anatomical CS and the TyG index in hypertensive individuals, and research has indicated that coronary artery function outweighs the effect of anatomy on clinical outcomes (10). In the literature, there is a lack of research that pays attention to the association between the TyG index and functional significant coronary artery stenosis in hypertensive individuals. Furthermore, no research has been done on the correlation between the TyG index, the fat attenuation index (FAI) of pericoronary adipose tissue (PCAT) and traditional plaque features in hypertensive patients.

Therefore, in this study our goal consists of: (1) exploring the connection between functional significant stenosis and TyG index in hypertensive individuals; (2) studying the association between pericoronary adipose tissue index, hemodynamics, plaque characteristics, and TyG index in patients affected by hypertension.

## Materials and methods

### Study population

**Figure 1:**
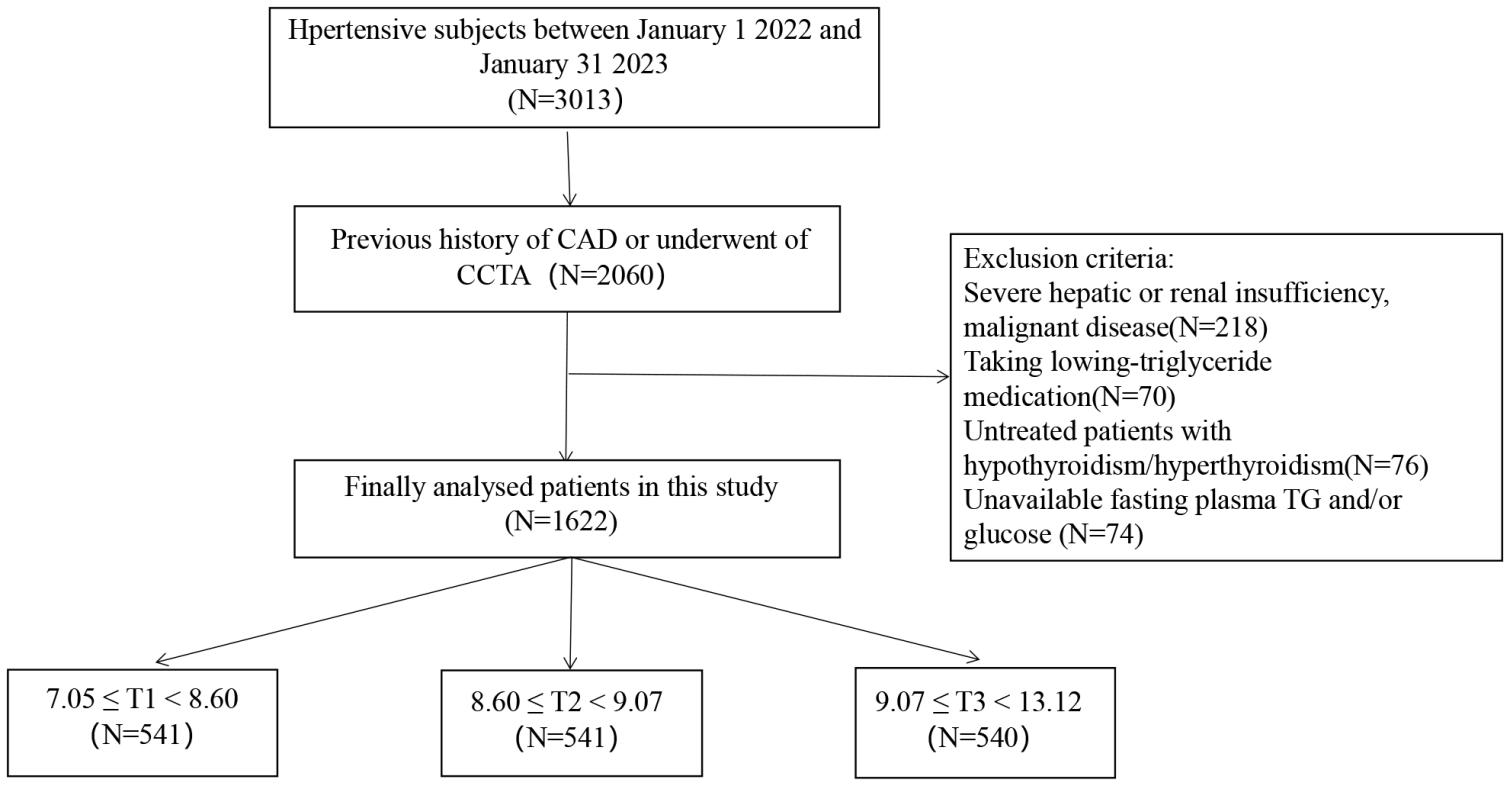
Study flow chart. CAD, coronary artery disease; CCTA, coronary computed tomography angiography; TG, triglyceride; T, tertile.

The research was cross-sectional and observational. This research included consecutive subjects with hypertension who met the 2017 ACC/AHA guidelines (3) and underwent CCTA in the Fourth Affiliated Hospital of Harbin Medical University during January 2022 and January 2023 due to typical or atypical chest pain. Exclusion criteria included inaccessible fasting glucose and triglyceride (TG) values, malignant disease, uncontrolled hyperthyroidism or hypothyroidism, and significant hepatic or renal failure. Moreover, those on low-triglyceride medications were excluded. Finally, 1622 eligible participants were enrolled for analysis in our research (a detailed flow chart is displayed in **Figure 1**).

This study program is executed pursuant to the principles delineated in the Helsinki Declaration, and it has been endorsed by our institutional committee in conformity with local ethical norms (Approved No. of Ethics Committee: 2023–41). Given that this is a retroactive inquiry, the patient’s informed consent was discarded.

### Data acquisition

From each patient’s medical history, the following details were gathered: age, sex, smoking, drinking, and history of prescription use. All participants had their fasting venous blood samples taken early in the morning. Laboratory analysis included measurements of creatinine, uric acid (UA), fasting blood glucose (FBG), total cholesterol (TC), high density lipoprotein (HDL-C), low density lipoprotein (LDL-C), triglyceride (TG) and other biochemical indices. The TyG index was calculated by ln (fasting TG [mg/dL] * fasting glucose [mg/dL]/2) (11). The formula for calculating body mass index (BMI) was weight divided by square height (19). Hyperlipidemia was characterized as TG ≥ 2.3 mmol/L, LDL-C ≥ 4.1 mmol/L, HDL-C ≤ 1.0 mmol/L or TC ≥ 6.2 mmol/L (20). Glycosylated hemoglobin ≥ 6.5% and FBG ≥ 7.0 mmol/L or random blood glucose > 11.1 mmol/L were the diagnostic criteria for diabetic mellitus (DM) (21).

### CT image acquisition

Every scan was conducted by a 320-detector-row CT scanner (Aquilion ONE, Toshiba, Tokyo, Japan), with noncontrast chest CT scans at admission and coronary CTA scans during hospital stays. Fast heart rate individuals were given oral metoprolol (25–75 mg) at baseline one hour before the test.

It was necessary to use a dual-channel high-pressure syringe to inject 60–80 mL of nonionic iodine contrast agent. Coronary CTA images were taken using prospective ECG triggering. The settings used in our experiment for acquisition and reconstruction were the rack rotation speed at 275 ms/time, tube potential at 120 kV, automatic tube current modification based on patient BMI, and 0.5 mm slice increments. Two expert investigators in cardiovascular imaging reviewed the collected imaging data.

### Visual recognition of adverse plaque characteristics

Segment analysis was utilized to detect adverse plaque characteristics (HRPCs) consisting of spotty calcification, positive remodeling (PR), low-attenuation plaque (LAPs), and the napkin ring sign (NRS). Low-attenuation plaque has an attenuation density of less than 30 Hounsfield Units (HU), while coronary artery wall calcification with a diameter of less than 3 mm was classified as spotty calcification. An outer vessel diameter greater than 10% of the mean diameter of the segments immediately proximal and distal to the plaque was considered positive remodeling. The ‘napkin ring’ sign, which consists of a high-attenuation outer rim surrounding a low-attenuation core region (22). High-risk plaques (HRPs) were defined as those that met two or more of those criteria (23).

### Pericoronary fat attenuation index acquisition

The PCAT was quantitatively quantified using specialized software (Shukun Technology).

For all voxels in the range of -190 to -30 Hounsfield units (HU), PCAT is defined as the radial distance from the vessel wall equal to the vessel diameter. The average CT attenuation of PCAT (HU) is quantified by the FAI (24). We analyzed PCAT in the 10- to 50-mm segment proximal to the right coronary artery (RCA). The CT measurement of FAI is fully automated and requires a small amount of additional manual optimization.

### Analysis of hemodynamic parameters of CCTA

A computational fluid dynamics-based software model (Shukun Technology) was utilized for the hemodynamic study. The software is used to segment the three-dimensional coronary artery model semiautomatically, and the FFR_CT_ value of any stenosis at the distal end of each vessel is recorded. When there are numerous stenoses, the distal FFR_CT_ value of the most severe lesion is used. If there was no stenosis, the FFR_CT_ value of the middle segment of the vessel was recorded.

### Statistical analyses

Datasets were examined using R version 4.2.2 and SPSS (25.0, IBM Corp, New York).

Normally distributed data are represented by the means ± standard deviations and were contrasted with an analysis of variance, or *T* test. Nonparametric data, which are presented as medians (interquartile ranges), were analyzed by the Mann−Whitney U test or the Kruskal−Wallis H test. Categorical variables were examined with the Chi-squared test and are described as percentages. The study utilized logistic regression analysis to examine the connection between FFR_CT_ and the TyG index, incorporating confounding variables into the regression equation to account for potential baseline data impacts. Model 1 was modified for sex, BMI, and age, and Model 2 was modified for drinking, smoking, hyperlipidemia, DM, creatine, UA, TG, FBG, ALT, HDL-C, and ApoA, which rested on Model 1. Model 3 was modified for NRS, LAPs, PR, spotty calcification, HRPs, coronary artery calcium score (CACS), and FAI, which rested on Model 2. The nonlinear connection with the TyG index and FFR_CT_ ≤ 0.80 was investigated using restricted cubic splines. The independent variables causing the increase in the TyG index were found using both univariate and multivariate linear regression. A *p* value of < 0.05 is deemed statistically significant.

## Results

### Baseline characteristics of the patient cohort

This study examined 1622 hypertensive patients, revealing that males constituted 45.07% of the population and 53.58% had hyperlipidemia. In all, 554 (34.16%) individuals had myocardial ischemia (FFR_CT_ ≤ 0.80). Within the study population, the patients’ average age was 62.43 ± 10.12 years. The median TyG index was 8.84 (IQR 8.48-9.22). The TyG index tertile was utilized to divide the available participants into three groups in **Table 1**.

**Table 1:** Clinical and biological characteristics according to TyG index tertiles.

Variables		TyG index level			*p* value
		Group T1 (N = 541)	Group T2 (N = 541)	Group T3 (N = 540)	
Demographics					
Age (years)		63.18 ± 9.64	63.35 ± 10.04	60.77 ± 10.48	< 0.001
Male, n (%)		233 (43.07%)	226 (41.77%)	272 (50.37%)	0.009
BMI (kg/m2)		24.69 (22.29, 26.8)	25.39 (23.14, 27.7)	26.33 (24.22, 28.7)	< 0.001
Hyperlipidemia, n (%)		164 (30.31%)	267 (49.35%)	438 (81.11%)	< 0.001
Smoking, n (%)		80 (14.79%)	73 (13.49%)	112 (20.74%)	0.003
Drinking, n (%)		50 (9.24%)	47 (8.69%)	69 (12.78%)	0.055
DM, n (%)		49 (9.06%)	109 (20.15%)	283 (52.41%)	< 0.001
Laboratory indicators					
TC (mmol/L)		4.75 (4.01, 5.47)	5.09 (4.45, 5.85)	5.31 (4.56, 6.17)	< 0.001
TG (mmol/L)		0.85 (1.02, 1.21)	1.58(1.41, 1.8)	2.43 (1.97, 3.16)	< 0.001
HDL-C (mmol/L)		1.21 (1.04, 1.39)	1.09 (0.95, 1.25)	1.02 (0.91, 1.16)	< 0.001
LDL-C (mmol/L)		2.72 (2.15, 3.27)	3.02 (2.47, 3.56)	2.92 (2.34, 3.47)	< 0.001
ApoA (g/L)		1.29 (1.15, 1.44)	1.27 (1.13, 1.4)	1.24 (1.13, 1.39)	0.013
ApoB (g/L)		0.89 (0.73, 1.05)	1.01 (0.85, 1.16)	1.04 (0.87, 1.21)	< 0.001
Lp(a) (mg/dl)		12.20 (7.00, 23.75)	11.90 (7.10, 24.50)	10.30 (5.70, 20.95)	< 0.001
FBG (mmol/L)		4.91 (4.62, 5.37)	5.33 (4.88, 5.97)	6.35 (5.29, 8.86)	< 0.001
Creatinine (μmmol/L)		67.00 (58.60, 78.40)	67.60 (58.95,79.25)	69.00 (58.60, 8 1.10)	0.475
UA (μmmol/L)		314(258.70, 370)	331 (277.05, 397.7)	350.7 (294, 420.7)	< 0.001
AST (U/L)		21 (18, 25)	21 (18, 26)	22 (19, 28)	< 0.001
ALT (U/L)		17 (13, 24)	19 (14, 26)	22 (16, 32)	< 0.001
TyG index		8.35 (8.16, 8.49)	8.84 (8.73, 8.94)	9.41 (9.22, 9.79)	< 0.001
Drug therapy					
ACE inhibitor or ARB, n (%)		135 (24.95%)	143 (26.43%)	139 (25.74%)	0.856
Calcium channel blocker, n (%)		240 (44.36%)	253 (46.77%)	248 (45.93%)	0.723
Other antihypertensive drugs, n (%)		32 (5.91%)	33 (6.10%)	32 (5.93%)	0.990
CACS		13.86 (0.00, 139.7)	26.12 (0.00, 183.0)	43.68 (0.00, 300.63)	< 0.001
High-risk plaque characteristics					
Positive remodeling, n (%)		59 (10.91%)	100 (18.48%)	112 (20.74%)	< 0.001
Spotty calcification, n (%)		92 (17.01%)	116 (21.44%)	160 (29.63%)	< 0.001
Low attenuation plaque, n (%)		41 (7.58%)	57 (10.54%)	98 (18.15%)	< 0.001
Napkin-ring sign, n (%)		24 (4.44%)	25 (4.62%)	49 (9.07%)	0.001
High-risk plaque, n (%)		64 (11.83%)	78 (14.42%)	136 (25.19%)	< 0.001
FAI (HU)		-88 (-95, -81)	-86 (-93, -78)	-82 (-88, -76)	< 0.001
FFR_CT_		0.9 (0.82, 0.93)	0.88 (0.77, 0.92)	0.82 (0.73, 0.88)	< 0.001
FFR_CT_ ≤ 0.80		115 (21.26%)	182 (33.64%)	257 (47.59%)	< 0.001

Data are presented as mean ± SD, median [25th–75th percentiles] or n ± (%).

BMI, body mass index; DM, diabetes mellitus; FBG, fasting blood glucose; TG, triglyceride; TC, total cholesterol; HDL-C, high-density lipoprotein cholesterol; LDL-C, low-density lipoprotein cholesterol; Apo, Apolipoprotein; Lp(a), lipoprotein(a); UA, uric acid; AST, aspartate aminotransferase; ALT, alanine aminotransferase; TyG, triglyceride glucose; CACS, coronary artery calcium score; FAI, fat attenuation index; FFR_CT_, CT-derived fractional flow reserve.

Individuals with the highest TyG index were male, younger, had a higher BMI, smoking, and had a history of diabetes and hyperlipidemia (*p* < 0.05). The TyG index seemed to have an inverse relationship with HDL-C, apolipoprotein A (ApoA), lipoprotein (A) [Lp (A)], FFR_CT_ and a positive relationship with TG, FBG, TC, apolipoprotein B (ApoB), UA, alanine aminotransferase (ALT), and CACS (*p* < 0.05). Likewise, we noticed a noticeable rise in the incidence of FFR_CT_ ≤ 0.80 as the TyG index rose. (*p* < 0.001).

There were notable variations across the three groups, and the FAI rose as the TyG index value increased (*p* < 0.001). Group T3 had a greater occurrence of HRPs and HRPCs, which comprise spotty calcification, PR, LAPs, and NRS, in comparison to Groups T1 or T2 (*p* < 0.05).

### Baseline characteristics of groups categorized with FFR_CT_

The baseline features of the subjects are illustrated in **Table 2**. Compared with the control group, the majority of the subjects with FFRCT ≤ 0.80 were described as follows: old, male, smoking, drinking, and a history of hyperlipidemia and DM (*p* < 0.05). The FFR_CT_ ≤ 0.80 group displayed a greater TG, ApoB, FBG, creatine, UA, and TyG index but a lower ApoA and HDL-C. The median FFR_CT_ values for the FFR_CT_ > 0.80 and FFR_CT_ ≤ 0.80 groups were 0.90 and 0.71, correspondingly. HRPs and HRPCs, including spotty calcification, PR, NRS, and LAPs, were more likely to occur in individuals with an FFR_CT_ ≤ 0.80 (*p* < 0.05).

**Table 2:** Clinical characteristics of patients stratified according to FFR_CT_.

Variables	Total (N = 1622)	FFR_CT_ ≤ 0.80 (N = 554)	FFR_CT_ > 0.80 (N = 1068)	*p* value
Demographics				
Age (years)	62.43 ± 10.12	63.42 ± 10.29	61.93 ± 10.00	0.005
Male, n (%)	731 (45.07%)	301 (54.33%)	430 (40.26%)	< 0.001
BMI (kg/m2)	25.47 (23.19, 27.73)	25.95 (23.44, 27.89)	25.35 (23.07,2 7.68)	0.016
Hyperlipidemia, n (%)	869 (53.58%)	348 (62.82%)	521 (48.78%)	< 0.001
Smoking, n (%)	265 (16.34%)	132 (23.81%)	133 (12.45%)	< 0.001
Drinking, n (%)	166 (10.23%)	75 (13.54%)	91 (8.52%)	0.002
DM, n (%)	441 (27.19%)	225 (40.61%)	216 (20.22%)	< 0.001
Laboratory indicators				
TC (mmol/L)	5.04 (4.32, 5.83)	5.04 (4.27, 5.85)	5.04 (4.33, 5.83)	0.857
TG (mmol/L)	1.52 (1.14, 2.09)	1.67 (1.28, 2.31)	1.45 (1.07, 1.96)	< 0.001
HDL-C (mmol/L)	1.09 (0.95, 1.28)	1.05 (0.92, 1.22)	1.11 (0.97, 1.31)	< 0.001
LDL-C (mmol/L)	2.88 (2.3, 3.44)	2.88 (2.26, 3.47)	2.89 (2.32, 3.42)	0.929
ApoA (g/L)	1.27 (1.14, 1.41)	1.24 (1.12, 1.38)	1.28 (1.15, 1.43)	< 0.001
ApoB (g/L)	0.98 (0.81, 1.14)	1.00 (0.82, 1.16)	0.97 (0.81, 1.13)	0.023
Lp(a) (mg/dl)	11.40 (6.60, 23.00)	11.20 (6.50, 23.90)	11.45 (6.60, 22.30)	0.829
FBG (mmol/L)	5.33 (4.84, 6.27)	5.83 (5.00, 7.59)	5.19 (4.77, 5.86)	< 0.001
Creatinine (μmmol/L)	68.1 (58.6, 79.7)	70.00 (60.58, 80.63)	67.05 (58.10, 79.00)	0.006
UA (μmmol/L)	331.05 (275.15, 397.05)	344.25 (287.08, 405.48)	322.70 (270.38, 393.18)	< 0.001
AST (U/L)	21 (18, 26)	22 (18, 27)	21 (18, 26)	0.448
ALT (U/L)	20 (15, 27)	20 (15, 28)	19 (14, 27)	0.090
TyG index	8.84 (8.48, 9.22)	8.99 (8.67, 9.44)	8.76 (8.40, 9.10)	< 0.001
Drug therapy				
ACE inhibitor or ARB, n (%)	417 (25.71%)	156 (28.16%)	261 (24.44%)	0.104
Calcium channel blocker, n (%)	741 (45.68%)	246 (44.40%)	495 (46.35%)	0.456
Other antihypertensive drugs, n (%)	97 (5.98%)	32 (5.78%)	65 (6.09%)	0.803
CACS	27.69 (0.00, 197.08)	152.17 (19.66, 523.13)	8.05 (0.00, 86.06)	< 0.001
High-risk plaque characteristics				
Positive remodeling, n (%)	271 (16.71%)	132 (23.83%)	139 (13.01%)	< 0.001
Spotty calcification, n (%)	368 (22.69%)	155 (27.98%)	213 (19.94%)	< 0.001
Low attenuation plaque, n (%)	196 (12.08%)	112 (20.22%)	84 (7.87%)	< 0.001
Napkin-ring sign, n (%)	98 (6.04%)	60 (10.83%)	38 (3.56%)	< 0.001
High-risk plaque, n (%)	278 (17.14%)	152 (27.44%)	126 (11.80%)	< 0.001
FAI (HU)	-85 (-92, -78)	-84 (-91, -78)	-86 (-93, -79)	0.002
FFR_CT_	0.87 (0.76, 0.92)	0.71 (0.63, 0.76)	0.90 (0.87, 0.93)	< 0.001

Data are presented as mean ± SD, median [25th–75th percentiles] or n ± (%).

BMI, body mass index; DM, diabetes mellitus; FBG, fasting blood glucose; TG, triglyceride; TC, total cholesterol; HDL-C, high-density lipoprotein cholesterol; LDL-C, low-density lipoprotein cholesterol; Apo, Apolipoprotein; Lp(a), lipoprotein(a); UA, uric acid; AST, aspartate aminotransferase; ALT, alanine aminotransferase; TyG, triglyceride glucose; CACS, coronary artery calcium score; FAI, fat attenuation index; FFR_CT_, CT-derived fractional flow reserve.

Moreover, in contrast to the FFR_CT_ > 0.80 group, the FFR_CT_ ≤ 0.80 group had a significantly greater proportion of HRPCs (**Additional Table 1**). The percentage of HRPCs varied significantly between the two groups (*p* < 0.001; Additional **Figure 1**). Lesions with ≥ 2 HRPCs appeared in 26.71% and 11.80% of the two groups, respectfully (**Additional Table 1**, **Additional Figure 1**). In contrast to the FFR_CT_ > 0.80 group, the FAI and CACS values in the FFR_CT_ < 0.80 group were greater (*p* = 0.002, *p* < 0.001).

### Association between the TyG index and FFR_CT_

**Figure 2:**
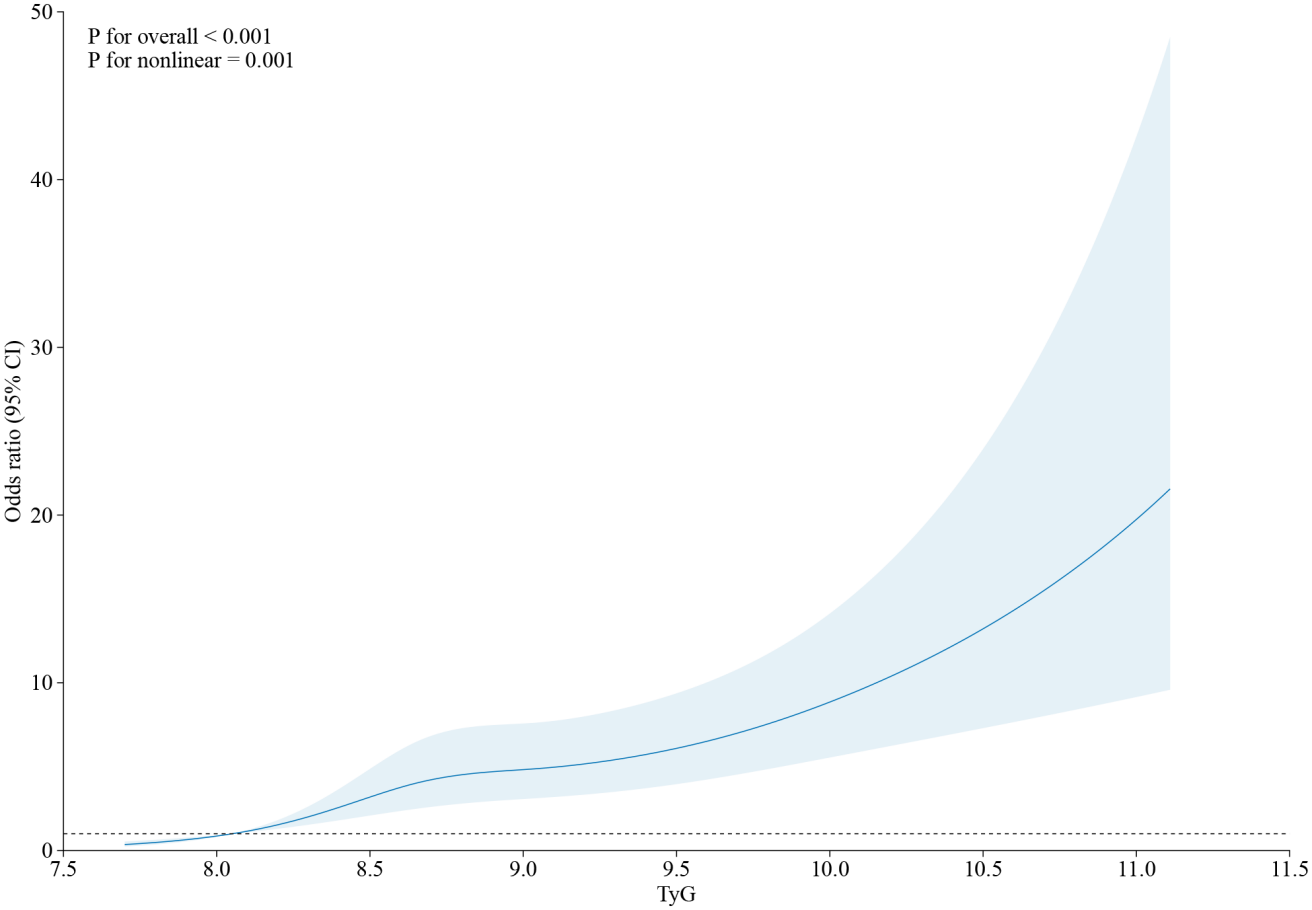
The restricted cubic spline of TyG index and the incidence of FFR_CT_ ≤ 0.80. Odds ratios and 95% CIs derived from restricted cubic spline regression, with knots placed at the 5th, 35th, 65th, and 95th percentiles of the distribution of TyG index. OR, odds ratio; CI, confidence interval; CAD, coronary artery disease; TyG index, triglyceride glucose index; RCS, restricted cubic spline.

The FFR_CT_ < 0.80 and the TyG index showed a positive connection in an unadjusted logistic regression model. Following the adjustment for other variables, there was still a separate correlation between the TyG index and FFR_CT_ ≤ 0.80 in Model 1, Model 2, and Model 3 (OR = 2.537, 95% CI 2.100–3.065, *p* < 0.001; OR = 2.135, 95% CI 1.403–3.247, *p* < 0.001; OR = 1.718, 95% CI 1.097–2.690, *p* = 0.018) (**Table 3**). The TyG index exhibited a nonlinear correlation with an FFR_CT_ ≤ 0.80 in its continuous range (*p* for nonlinear = 0.001) (**Figure 2**).

**Table 3:** Association of TyG index with the risk of FFR_CT_ ≤ 0.80 in logistic regression models.

	OR	95% CI	*p* value
Unadjusted model			
TyG, per 1-unit increase	2.395	2.000-2.867	< 0.001
Model 1			
TyG, per 1-unit increase	2.537	2.100-3.065	< 0.001
Model 2			
TyG, per 1-unit increase	2.135	1.403-3.247	< 0.001
Model 3			
TyG, per 1-unit increase	1.718	1.097-2.690	0.018

Model 1: adjusted for age, sex, and BMI.

Model 2: adjusted for age, sex, BMI, drinking, smoking, Hyperlipidemia, DM, creatine, UA, TG, FBG, ALT, HDL-C, ApoA.

Model 3: adjusted for age, sex, BMI, drinking, smoking, Hyperlipidemia, DM, creatine, UA, TG, FBG ALT, HDL-C, ApoA, Positive remodeling, Spotty calcification, Low attenuation plaque, Napkin-ring sign, High-risk plaque, FAI, CACS.

TyG, triglyceride glucose; OR, odds ratio; CI, confidence interval; BMI, body mass index; DM, diabetes mellitus; FBG, fasting blood glucose; TG, triglyceride; HDL-C, high-density lipoprotein cholesterol; Apo, Apolipoprotein; UA, uric acid; ALT, alanine aminotransferase; CACS, coronary artery calcium score; FAI, fat attenuation index; FFR_CT_, CT-derived fractional flow reserve.

### TyG index and FFR_CT_ relationships in specific subgroups

**Figure 3:**
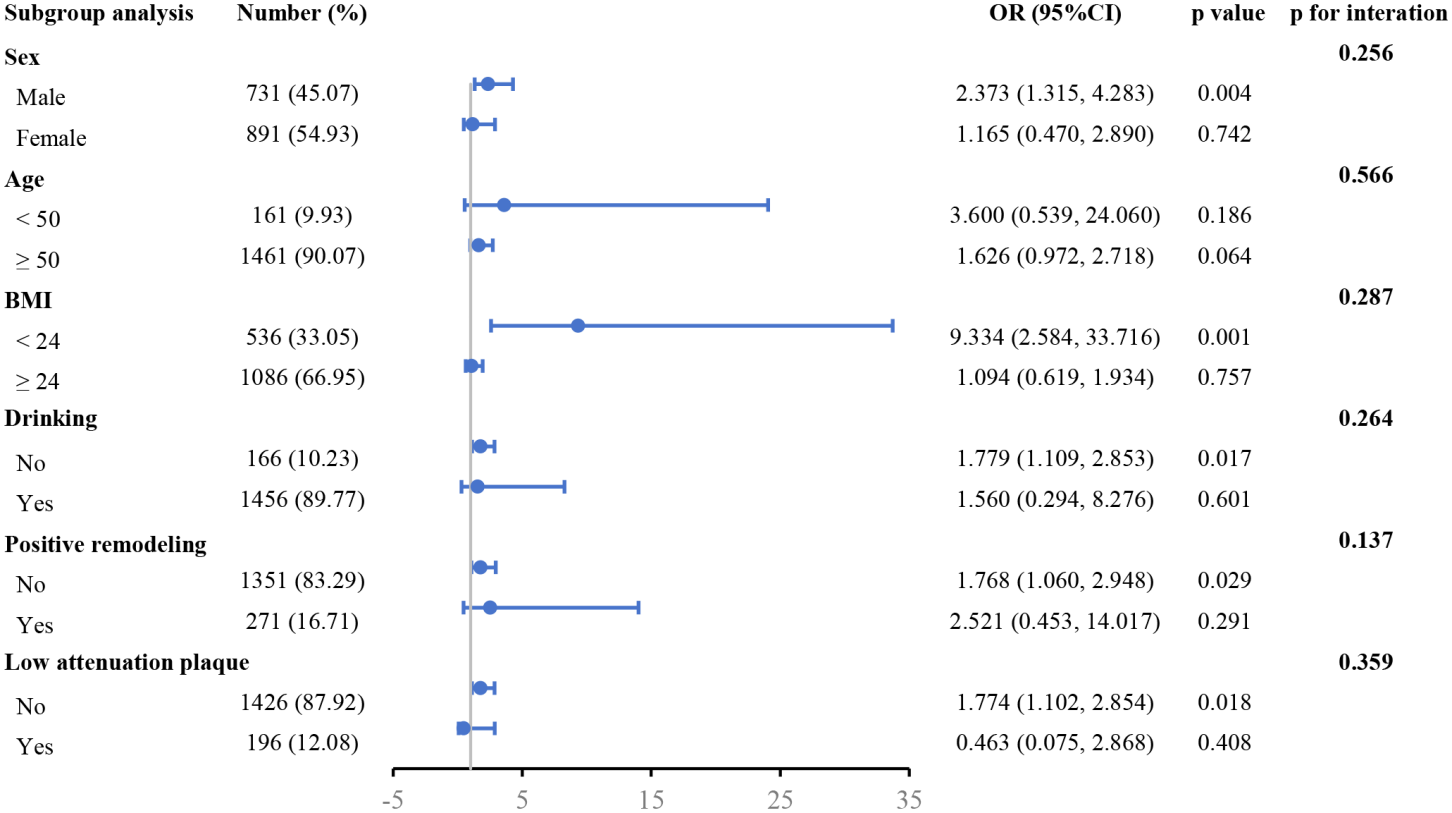
Forest plot investigating the association between the TyG index and with the risk of FFR_CT_ ≤ 0.80 in different subgroups. TyG, triglyceride glucose; FFR_CT_, CT-derived fractional flow reserve; BMI, body mass index; OR, odds ratio; CI, confidence interval.

The correlation between FFR_CT_ ≤ 0.80 and the TyG index was evaluated across several subgroups (**Figure 3**). In the subgroups of males, BMI < 24 kg/m^2^, no drinking, no PR, and no LAPs, the positive correlation across FFR_CT_ ≤ 0.80 and the TyG index was more notable after controlling for other variables.

### CCTA imaging characteristics are linked to elevated TyG index levels

The variables linked to elevated TyG index levels were identified through the use of univariate and multivariate analyses (**Table 4**). Within the univariate analysis, a greater TyG index was closely linked with PR, spotty calcification, LAPs, NRS, HRPs, FAI, and FFR_CT_. A greater TyG index value was independently linked with FFR_CT,_ FAI, PR, and LAPs in the multivariate analysis (standardized regression coefficients: -0.051 [*p* < 0.001], 0.029 [*p* = 0.007], 0.029 [*p* = 0.027], and 0.026 [*p* = 0.046], separately).

**Table 4:** Uni- and multivariate linear regression analysis for the increased TyG index level.

Variables	Univariate Analysis			Multivariate Analysis		
TyG index	Unstandardized Coefficients	Standardized Coefficients	*p* value	Unstandardized Coefficients	Standardized Coefficients	*p* value
Male, n (%)	0.096	0.077	0.002	-0.091	-0.073	< 0.001
Age (years)	-0.007	-0.120	< 0.001	-0.002	-0.039	0.001
BMI (kg/m2)	0.033	0.205	< 0.001	0.005	0.034	0.003
Smoking, n (%)	0.181	0.108	< 0.001	0.004	0.003	0.824
Drinking, n (%)	0.127	0.062	0.013	0.025	0.012	0.285
UA (μmmol/L)	0.001	0.219	< 0.001	0.000	0.064	< 0.001
AST (U/L)	0.003	0.086	0.001	-0.001	-0.023	0.054
ALT (U/L)	0.009	0.204	< 0.001	0.002	0.042	0.001
FBG (mmol/L)	0.162	0.579	< 0.001	0.104	0.374	< 0.001
TC (mmol/L)	0.142	0.275	< 0.001	-0.004	-0.008	0.831
TG (mmol/L)	0.276	0.730	< 0.001	0.199	0.524	< 0.001
HDL-C (mmol/L)	-0.748	-0.317	< 0.001	-0.780	-0.330	< 0.001
LDL-C (mmol/L)	0.045	0.066	0.008	0.041	0.060	0.057
ApoA (g/L)	-0.183	-0.065	0.009	0.888	0.314	< 0.001
ApoB (g/L)	0.069	0.106	< 0.001	0.007	0.011	0.300
Lp(a) (mg/dl)	-0.003	-0.084	0.001	0.000	-0.008	0.436
Hyperlipidemia, n (%)	0.548	0.440	< 0.001	0.183	0.147	< 0.001
DM, n (%)	0.594	0.426	< 0.001	0.083	0.060	< 0.001
Positive remodeling, n (%)	0.163	0.098	< 0.001	0.048	0.029	0.027
Spotty calcification, n (%)	0.119	0.080	0.001	0.034	0.023	0.133
Low attenuation plaque, n (%)	0.215	0.113	< 0.001	0.049	0.026	0.046
Napkin-ring sign, n (%)	0.129	0.050	0.046	-0.008	-0.003	0.775
High-risk plaque	0.172	0.105	< 0.001	-0.029	-0.018	0.377
FAI (HU)	0.012	0.190	< 0.001	0.002	0.029	0.007
FFR_CT_	-1.391	-0.264	< 0.001	-0.271	-0.051	< 0.001

BMI, body mass index; DM, diabetes mellitus; FBG, fasting blood glucose; TG, triglyceride; TC, total cholesterol; HDL-C, high-density lipoprotein cholesterol; LDL-C, low-density lipoprotein cholesterol; Apo, Apolipoprotein; Lp(a), lipoprotein(a); UA, uric acid; AST, aspartate aminotransferase; ALT, alanine aminotransferase; TyG, triglyceride glucose; FAI, fat attenuation index; FFR_CT_, CT-derived fractional flow reserve.

## Discussion

The following were the study’s main points: (1) In individuals with hypertension, the TyG index significantly affects the incidence of functionally significant stenosis. It implies that in identifying hypertensive patients at greater risk of myocardial ischemia and directing further detection and more intensive care, the TyG index may be useful. (2) The TyG index showed independent correlations with CT-derived fractional flow reserve (FFR_CT_), the fat attenuation index (FAI), low-attenuation plaque and positive remodeling in hypertensive patients.

The prominent risk cause for cardiovascular disease, hypertension is associated with worse outcomes. Compared to those without hypertension, subjects with hypertension exhibited more advanced coronary atherosclerosis by CCTA and a greater chance for major adverse cardiac events in the future (25). Therefore, identifying hypertensive individuals at increased risk for CAD is essential for guiding further diagnosis and early treatment. However, standard CCTA results related to coronary stenosis often overstate the degree of lesion-specific ischemia in CAD patients with severe grade stenosis (26, 27). FFR_CT_ is a noninvasive physiological detection method that is useful for precisely assessing lesion-specific ischemia and is superior to stenosis on coronary CT angiograms of both 50% or greater and greater than 70% in predicting clinical outcomes (28). Currently, a positive index of myocardial ischemia with FFRCT < 0.80 has been utilized to direct interventional treatment and assess prognosis (28, 29). Therefore, it is of clinical value to determine the risk factors influencing FFR_CT_ ≤ 0.80; they may have a bearing on early intervention.

It is commonly accepted that insulin resistance raises the risk of oxidative stress, inflammation, atherosclerosis, and endothelial dysfunction—all of which are prevalent in hypertension (30). The TyG index has already shown to be a faithful easy IR estimate indicator lately and is comparable to the gold standard for assessing IR, the euglycemic-hyperinsulinemic clamp technique (31, 32). Research has demonstrated that when predicting insulin resistance using the hyperglycemic clamp test, the TyG index outperformed the homeostasis model assessment of insulin resistance (HOMA-IR) index (11). Multiple analyses reported a clear connection between the onset of atherosclerotic cardiovascular disease and the TyG index (19, 31, 33). A correlation between the number and severity of coronary artery stenotic lesions and the TyG index was demonstrated by cohort studies (31). Furthermore, the TyG index is one method for forecasting the severity of coronary heart disease, independent of glucose metabolism (34). New research indicates that the brachia-ankle pulse wave velocity and the severity of coronary heart disease are strongly connected with the TyG index, which also relates to the risk of subclinical arteriosclerosis in hypertensive individuals (3, 33). However, no research has explored the connection between FFR_CT_ ≤ 0.80 and the TyG index in hypertensive patients. The TyG index and myocardial ischemia were shown to be correlated in this study, with a greater TyG index level indicating a better chance of FFR_CT_ ≤ 0.80. Treatment approaches that reduce the TyG index is possible helpful to reduce future myocardial ischemia in hypertensive patients. Furthermore, subgroup analysis findings indicated that FFR_CT_ ≤ 0.80 and the TyG index were likewise stable within various subgroups. Surprisingly, the subgroups of patients classified as men, BMI < 24 kg/m^2^, no drinking, no PR, and no LAPs showed a greater association with this connection. Although the precise process is unknown, it is a consideration that we must all take into account. Therefore, further exploration and research are needed in the future.

The TyG index and FFR_CT_ may be linked in hypertensive patients according to their assessment of IR status, although the precise mechanism behind this relationship is yet unknown. First, through oxidative stress, IR can impair the function of the coronary endothelium and cause inflammation, which leads to coronary artery dysfunction (21, 34). Second, IR is characterized by impaired vasodilation, microvascular disease, and atherosclerotic disease. Hemodynamic damage reduces the blood flow of the epicardial coronary artery (21, 35). Future research may be conducted to elucidate the connection between FFR_CT_ and the TyG index.

Additionally, this research also evaluated the correlation between the TyG index and adverse plaque characteristics (HRPCs) and the FAI in hypertensive patients. Damage to vascular endothelial cells brought on by IR can trigger an inflammatory reaction (23). It has been demonstrated that coronary artery inflammation inhibits fat formation in perivascular fat, thereby altering the attenuation of perivascular fat to CCTA (36). It has been demonstrated that the FAI index, which is based on the attenuation of pericoronary adipose tissue (PCAT) on CCTA, indicates the inflammation of the coronary artery wall. Around the proximal RCA, PCAT attenuation is a biomarker of worldwide coronary inflammation (29). This might explain why FAI and the TyG index are correlated. Simultaneously, our research demonstrates that individuals with an increased serum TyG index also frequently have high-risk plaques (HRPs) and typical HRPCs. This result is in line with other research (23, 37), showing a relationship between plaque stability and the TyG index. Thus, we hypothesize that serum TyG index level and plaque instability may be related.

Our research examined the correlation between FFR_CT_ ≤ 0.80 and the TyG index in hypertensive individuals and further investigated the links between the TyG index and FAI and HRPs. This research holds significant clinical utility. First, our research demonstrates that the TyG index is a risk indicator for FFR_CT_ ≤ 0.80 and that reducing the index may protect hypertensive patients from myocardial ischemia. Therefore, more interventional research is needed to ascertain if therapy targeted at lowering the quantity of IR as indicated by the TyG index improves myocardial ischemia in hypertensive individuals. Secondly, the TyG index is a blood indicator that may be used in real time to assess a patient’s coronary physiological function during follow-up since it is readily measured, accessible, and repeatable. In addition, we observed that an increased TyG index is more probably to be associated with HRPs and correspond to a higher FAI; this consideration will be helpful in further exploration of the effects of IR on plaque instability and coronary artery wall inflammation in hypertensive patients.

This study is restricted by some limitations. First, the study was cross-sectional and retrospective observational. We screened subjects in order. Some intrinsic variances do exist, though. Second, it has been reported that FFR_CT_ is consistent with fractional flow reserve (FFR) in invasive coronary angiography (ICA). However, FFR_CT_ is still not the best method for evaluating coronary artery disease-specific ischemia. Furthermore, our research did not include any quantitative evaluation of coronary artery plaque, which is an aspect that warrants analysis in future research.

## Conclusion

This study provides some proof that in hypertensive patients, the TyG index is independently connection with an elevated chance of FFR_CT_ ≤ 0.80. This will give useful data for early risk assessment and direct further detection efforts, and steer more focused treatment plans. Simultaneously, hypertensive patients who had a greater TyG index also had greater odds of HRPs, a lower FFR_CT_, and a higher FAI. Additionally, a separate correlation was seen between the TyG index and positive remodeling, low-attenuation plaque, FAI, and FFR_CT_.

## Data availability statement

The raw data supporting the conclusions of this article will be made available by the authors, without undue reservation.

## Ethics statement

The study protocol was approved by the Medical Ethics Committee of The Fourth Affiliated Hospital of Harbin Medical University (Approved No. of Ethics Committee: 2023–41) and conducted in accordance with the principles contained within the Declaration of Helsinki, but patient informed consent was waived because it was a retrospective study.

## Author contributions

SW: Conceptualization, Data curation, Formal analysis, Investigation, Methodology, Visualization, Writing – original draft, Writing – review & editing. ZS: Conceptualization, Data curation, Formal analysis, Investigation, Methodology, Visualization, Writing – original draft. HP: Conceptualization, Methodology, Project administration, Writing – original draft. TY: Data curation, Investigation, Project administration, Writing – original draft. LL: Conceptualization, Data curation, Methodology, Writing – original draft. JX: Conceptualization, Methodology, Writing – original draft. WW: Data curation, Investigation, Project administration, Visualization, Writing – review & editing. TZ: Conceptualization, Formal analysis, Funding acquisition, Investigation, Methodology, Project administration, Software, Supervision, Writing – original draft, Writing – review & editing.
