# Supplementary material for: Triglyceride glucose index is associated with functional coronary artery stenosis in hypertensive patients
Source: Front Endocrinol (Lausanne). 2024 Mar 25;15:1323722. doi: 10.3389/fendo.2024.1323722 (PMC10999614; doi:10.3389/fendo.2024.1323722)
Supplement: Supplementary file 1 [file DataSheet_1.docx]

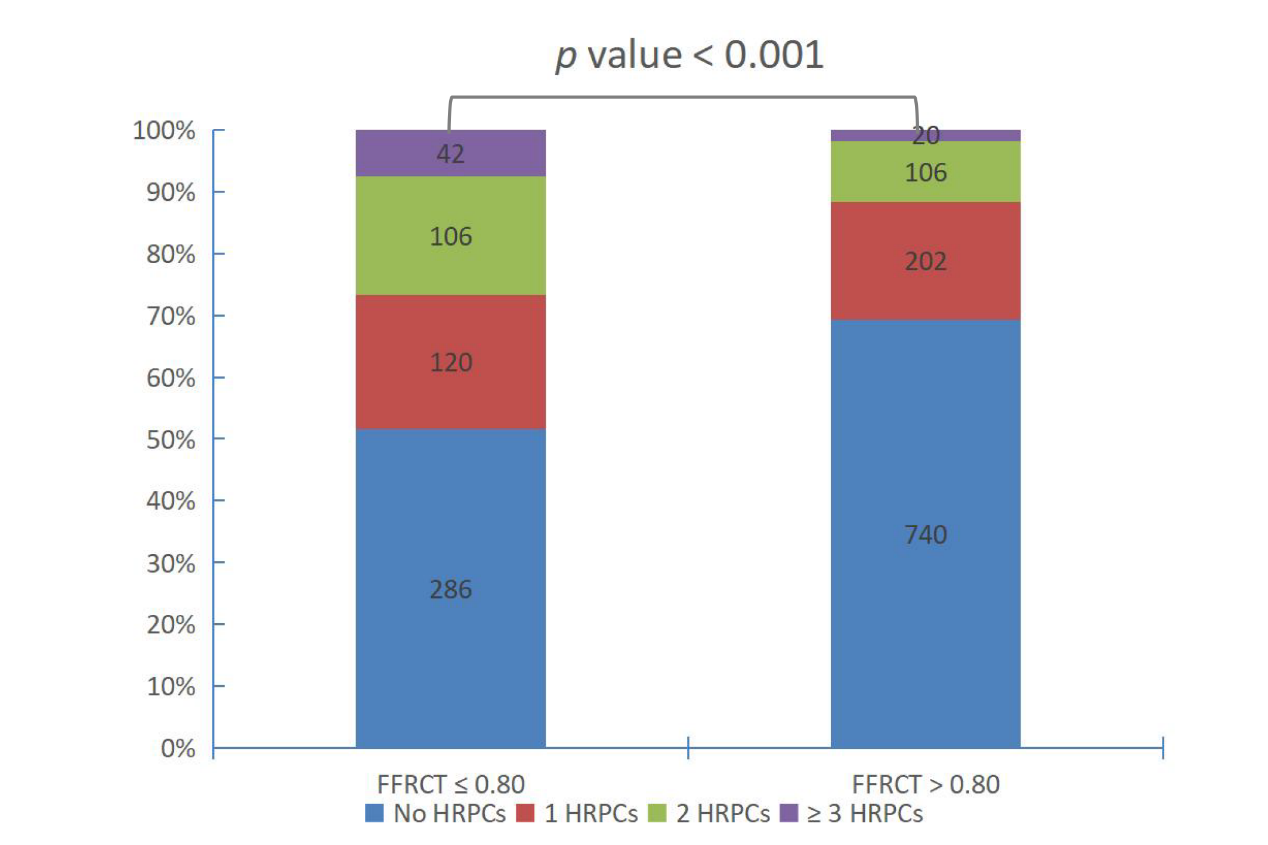


**Additional Figure 1**. Distribution of HRPCs According to FFR_CT_ Categories. The number of HRPCs was significantly different among different FFR_CT_ categories. HRPCs, adverse plaque characteristics, FFR_CT_, CT-derived fractional flow reserve.
