# Supplementary material for: Triglyceride glucose index is associated with functional coronary artery stenosis in hypertensive patients
Source: Front Endocrinol (Lausanne). 2024 Mar 25;15:1323722. doi: 10.3389/fendo.2024.1323722 (PMC10999614; doi:10.3389/fendo.2024.1323722)
Supplement: Supplementary file 2 [file DataSheet_2.docx]

**Additional Table 1** The number of HRPCs of patients stratified according to FFR_CT_.

| The number of HRPCs | Group | |
| --- | --- | --- |
|  | FFR_CT_ ≤ 0.80 | FFR_CT_ > 0.80 |
| No HRPCs, n (%) | 286 (51.62%) | 740 (69.29%) |
| 1 HRPCs, n (%) | 120 (21.66%) | 202 (18.91%) |
| 2 HRPCs, n (%) | 106 (19.13%) | 106 (9.93%) |
| ≥3 HRPCs, n (%) | 42 (7.58%) | 20 (1.87%) |

HRPCs, High-risk plaque characteristics; FFR_CT_, CT-derived fractional flow reserve
